# *Mycobacterium tuberculosis* Subverts the TLR-2 - MyD88 Pathway to Facilitate Its Translocation into the Cytosol

**DOI:** 10.1371/journal.pone.0086886

**Published:** 2014-01-27

**Authors:** Md. Aejazur Rahman, Parveen Sobia, Neeta Gupta, Luc Van Kaer, Gobardhan Das

**Affiliations:** 1 School of Laboratory Medicine and Medical Science, University of KwaZulu-Natal, Durban, South Africa; 2 Immunology Group, International Center for Genetic Engineering and Biotechnology, Aruna Asaf Ali Marg, New Delhi, India; 3 Department of Pathology, Microbiology and Immunology, Vanderbilt University School of Medicine, Nashville, Tennessee, United States of America; Tulane University, United States of America

## Abstract

*Mycobacterium tuberculosis* (*M.tb*) has evolved mechanisms to evade its destruction in phagolysosomes, where it successfully survives and replicates within phagocytes. Recent studies have shown that virulent strains of *M.tb* can translocate from the phagosome into the cytosol of dendritic cells (DC). The molecular mechanisms by which virulent *M.tb* strains can escape the phagosome remain unknown. Here we show that the virulent *M.tb* strain H37Rv, but not the vaccine strain Bacille Calmette-Guérin (BCG), escapes from the phagolysosome and enters the cytosol by interfering with the TLR-2-MyD88 signaling pathway. Using H37Rv mutants, we further demonstrate that the region of difference-1 (RD-1) locus and ESAT-6, a gene within the RD-1 locus, play an important role in the capacity of *M.tb* to migrate from the phagosome to the cytosol of macrophages. H37Rv, BCG, H37RvΔRD1, and H37RvΔESAT6 were able to translocate to the cytosol in macrophages derived from TLR-2- and MyD88-deficient animals, whereas only virulent H37Rv was able to enter the cytosol in macrophages from wild type mice. Therefore, signaling through the TLR-2–MyD88 pathway in macrophages plays an important role in confining *M.tb* within phagolysomes. Virulent strains of *M.tb* have evolved mechanisms to subvert this pathway, thus facilitating their translocation to the cytosol and to escape the toxic microenvironment of the phagosome or phagolysosome.

## Introduction

Phagocytes, the primary innate immune cells, engulf microorganisms in phagosomes, which later fuse with lysosomes. The acidic environment of the phagolysosome degrades the harbored organisms, and this process makes antigens available for priming of T cell responses. *Mycobacterium tuberculosis* (*M.tb*), the causative agent of tuberculosis (TB), evolved mechanisms for inhibiting phagosome maturation [1], [2] and to neutralize the acidic environment of the phagolysosomal compartment [3], [4] to ensure its unhindered survival and replication within phagocytes. It has been widely accepted that *M.tb* remains within phagosomes of macrophages by inhibiting phagosome acidification. However, recent studies have demonstrated that *M.tb* and *M. leprae* successfully translocate to the cytosol from the phagolysosome in dendritic cells (DCs) [5]. This observation is consistent with studies of *M. marinum* infection, which also translocate to the cytosol [6]–[8]. ESAT-6, a gene product of region of difference 1 (RD-1), has been shown to play a key role in this process, and thus contribute to bacterial virulence [8]. *M. bovis* vaccine strain Bacille Calmette-Guérin (BCG), which lacks the RD-1 region, is unable to translocate to the cytosol and is avirulent [9]. Therefore, cytosolic escape could be a potential mechanism of virulence exerted by the proteins encoded within the RD-1 region [8]. The ESAT-6/CFP-10 complex has been shown to play an important role in release and spread of *M.tb* to neighbouring cells [10] but the mechanism employed by *M.tb* to translocate from the phagolysosome to the cytosol remains elusive [11]. Previous studies have indicated that the Toll-like receptor (TLR) pathway plays an important role in phagosome maturation or phagolysosome formation [12]. Thus, fusion of phagosomes with lysosomes is hindered in macrophages isolated from MyD88-deficient mice [12]. However, some investigators found that there is no apparent role of innate TLR-mediated signals in phagolysosomal maturation [13]. Thus, we examined if TLR signaling has a role in intracellular translocation of *M.tb* from phagolysosomes to the cytosol in macrophages.

Here, we show that the virulent *M.tb* strain H37Rv escapes the phagolysosomal or endosomal compartment and translocates to the cytosol, whereas avirulent BCG remained in phagolysosomes of macrophages derived from wild type C57BL/6 mice. Interestingly, both H37Rv and BCG translocated to the cytosol of macrophages isolated from TLR-2^−/−^ and MyD88^−/−^ mice. Therefore, TLR-2-MyD88 signaling plays an important role in confining pathogens within the phagolysosomal compartment. We further found that ESAT-6 (ΔESAT-6) and RD-1 (ΔRD-1) mutants of H37Rv were unable to translocate to the cytosol, suggesting that the ESAT-6/CFP-10 complex plays an important role in the escape of virulent *M.tb* from the phagolysosome to the cytosol. Interestingly, both virulent (H37Rv) and avirulent (BCG, H37RvΔRD-1, and H37RvΔESAT-6) strains were able to enter the cytosol of macrophages isolated from TLR-2^−/−^ or MyD88^−/−^ mice. We also observed that the virulent strain H37Rv but not its ΔESAT-6 variant inhibited the expression of MyD88 and TRAF-6 in macrophages. Therefore, in the absence of TLR signaling, the phagosomal compartment is unable to confine the harbored organisms. *M.tb* exploits this process by down regulating TLR-mediated signals, which permits it to translocate to the cytosol. This represents a novel immune evasion mechanism adopted by *M.tb* to avoid immune recognition.

## Materials and Methods

### Ethics statement

All animal experiments were conducted in accordance with guidelines approved by the Institutional Animals Ethics Committee of ICGEB, New Delhi, India and Department of Biotechnology (DBT), Government of India, that also specifically approved the study. All mice used for these experiments were ethically sacrificed by asphyxiation in carbon dioxide according to institutional and DBT regulations.

### Mice and bacterial cultures

C57BL/6 mice were obtained from the animal facility at the International Center for Genetic Engineering and Biotechnology (ICGEB, New Delhi, India). MyD88 and TLR-2 knockout mice on the C57BL/6 background were a kind gift from Prof. Ruslan Medzhitov (Yale University School of Medicine, New Haven, USA). These animals were maintained and bred in the specific pathogen-free animal facility of ICGEB. *M. tuberculosis* H37Rv, *M. bovis* BCG, H37RvΔESAT6 and H37RvΔRD1 strains were a kind gift from Prof. David Sherman (Seattle Institute of biomedical research, Seattle, USA). These strains were grown in Middlebrook 7H9 broth (Difco, BD, USA) supplemented with Middlebrook albumin, dextrose, catalase (10% ADC, Difco, BD, USA) enrichment medium, 0.5% glycerol, and 0.1% Tween 80 until log phase (OD_600_∼0.6). Glycerol stocks were prepared for each culture and stored at −80°C. Tenfold serial dilutions of each strain were plated in duplicate onto Middlebrook 7H11 agar plates containing 10% OADC (oleic acid, albumin, dextrose and catalase) and incubated at 37°C for 21 days. Colonies on the plates were enumerated and colony-forming units (CFU) were calculated.

### Preparation of peritoneal macrophages

Mice were injected intraperitoneally with 2 ml of 4% thioglycollate (Brewer modified, BBL, BD, USA). Five days later, peritoneal exudate cells were isolated from the peritoneal cavity by washing with ice-cold RPMI1640 medium supplemented with 10% fetal bovine serum (FBS, Thermo Scientific HyClone, USA). Cells were cultured overnight at 37°C, 5% CO_2_ and washed with RPMI/10% FBS to remove non-adherent cells. Adherent monolayer cells were used as peritoneal macrophages. Peritoneal macrophages (5×10^5^ cells/ml) were cultured in RPMI/10% FBS and infected with *M*. *tuberculosis* H37Rv, *M. bovis* BCG, H37RvΔESAT6 or H37RvΔRD1 cultures (multiplicity of infection, 10∶1) after passing through a 26G needle (10–15×), incubated at 37°C for 4 hr. After 4 hr, cells were washed with fresh RPMI/10% FBS twice, and treated with 100 µg/ml gentamicin for 1 hr in medium to remove extracellular bacteria. After gentamicin treatment the culture medium was removed completely and fresh RPMI/10% FBS was added to the cells and cultured at 37°C and 5% CO_2_ for different time periods (0–96 hr).

### Confocal microscopy

Surface labelling of bacteria with FITC was done according to standard methods [14], just prior to infection. The bacteria were harvested, washed twice with PBS (pH 7.4) and re-suspended with 0.1 M sodium carbonate buffer (pH 9.5) containing FITC (1 mg/ml, Sigma-Aldrich, USA); the mixture was incubated for 30 min at room temperature with gentle shaking. The bacteria were collected by centrifugation, washed three times and suspended in RPMI1640 (without antibiotics), passed through a 26G needle and used as targets in assays of phagocytosis for confocal microscopy. Briefly, macrophages were plated in 12-well plates (∼1×10^6^/well) on glass cover slips and infected with FITC-labelled bacteria at a multiplicity of infection (MOI) of 5∶1 and the remainder of the infection protocol was then followed. After different time points the culture medium was removed from the wells, washed twice with PBS and cells were fixed with 2% paraformaldehyde in PBS, pH 7.4 for 20–30 min. After fixation cells were washed three times with PBS and cover slips were transferred into fresh plates and stored at 4°C until use. Cells were permeabilized with 0.2% Triton X-100 for 4 min, washed twice with PBS and incubated with 2% BSA for 5 min. Permeabilized macrophages were labelled with the following primary antibodies: rat anti-LAMP1 (CD107a, Cat. No. 553792; BD bioscience, USA) and mouse anti-Rab5 (Cat. No. 610282; BD bioscience, USA). Primary antibodies were detected with Alexa Fluor 594-conjugated rabbit anti-rat IgG (Cat. No. A-21211; Invitrogen, USA) or Alexa Fluor 594 conjugated goat anti-mouse IgG (Cat. No. A-11032; Invitrogen, USA) antibodies. Cover slips were mounted in Prolong Gold antifade reagent (Cat. No. P36934; Invitrogen, USA) and sealed using adhesives. Confocal microscopy images were acquired on a Nikon A-1R confocal microscope with a 60× (for statistical data) and 100× (for images) objectives and analyzed by using NIS element software. For each sample triplicate slides were prepared, and for each slide at least 4 different fields containing ∼10–15 cells were analyzed. Co-localization studies were performed after background correction using the NIS element software.

### Differential permeabilization of macrophages with digitonin

Differential permeabilization of macrophages with digitonin was essentially performed as previously described [15], [16]. Macrophages were isolated from the peritoneal cavity of C57BL/6, TLR2^−/−^ and MyD88^−/−^ mice and infected with different microorganisms (H37Rv, BCG, H37RvΔRD1 and H37RvΔESAT-6). Infected macrophages were washed 2× with KHM buffer (110 mM potassium acetate, 20 mM Hepes and 2 mM MgCl_2_), incubated with KHM buffer containing 25 µg/mL digitonin (D141, Sigma-Aldrich, USA) for 1 min at room temperature and then washed 1× with detergent-free KHM buffer. Macrophages were then incubated with antibody (anti-Mtb, ab905, Abcam) in KHM containing 3% BSA or with buffer alone at 32°C for 12 min, washed with PBS and fixed with 4% PFA. Macrophages that had been incubated with buffer alone were permeabilized with 0.2% Triton X-100 for 4 min and then incubated with antibody (anti-Mtb, ab905, Abcam). Bound antibody was visualized with Alexa 545-conjugated goat anti-rabbit IgG (Invitrogen, Carlsbad, CA) by incubating in 3% BSA in PBS for 30 min. Controls without primary antibodies showed no fluorescence from the secondary antibodies alone. Confocal microscopy images were acquired on a Nikon A-1R confocal microscope with 60× (for statistical data) and 100× (for images) objectives and analyzed by using NIS element software.

### Relative gene expression

RNA was prepared from macrophages infected with different *Mycobacterium* strains at 48 hr using TRIzol (Invitrogen, USA). A total of 1.5 µg of RNA was used for random hexamer-primed cDNA synthesis (Sensiscript RT kit; Qiagen, USA) according to the manufacturer's protocol. Real-time PCR was performed on a BIORAD Real-Time PCR System using IQ™ SYBR Green PCR master mix (BioRad, USA) and different sets of primers in triplicate. PCR steps were: 95°C for 5 min for 1 cycle, 95°C for 25 s, 53°C for 25 s, and 72°C for 1 min for 40 cycles. Fluorescence data were collected at each amplification step. The mRNA expression profiles were normalized with respect to GAPDH for each sample. Fold increase of each gene was calculated with respect to uninfected control at the same time point using the 2^−ΔΔCt^ method. Primers sequences were as follows: MyD88: forward, 5′-CCACCTGTAAAGGCTTCTCG-3′, reverse, 5′-CTAGAGCTGCTGGCCTTGTT-3′; TLR-2: forward, 5′CCAAAGAGCTCGTAGCATCC-3′, reverse, 5′-AGGGGCTTCACTTCTCTGCT-3′; TRAF6: forward, 5′-ATTTCATTGTCAACTGGGCA-3′, reverse, 5′-TGAGTGTCCCATCTGCTTGA-3′; TIRAP: forward, 5′-TGGCCTTCAACAGTCTTCCT-3′, reverse, 5′-CAGATCCCGAGTCCTACCAA-3′; IRAK4: forward, 5′-ACAGCTTCCTAAGGATCCCC-3′, reverse, 5′-GATCGCCTTGTCCAGGAAG-3′.

## Results

### Virulent *M.tb*, but not avirulent BCG, translocates from the phagolysosome to the cytosol in macrophages

**Figure 1:**
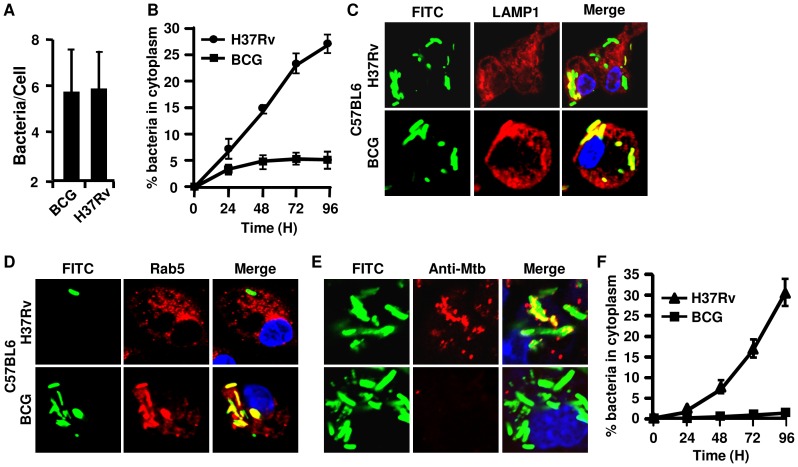
*Mycobacterium tuberculosis* H37Rv, but not *M. bovis* BCG, translocates into the cytosol of murine peritoneal macrophages. Bacteria were labelled green with FITC and used to infect peritoneal macrophages from C57BL/6 mice at an MOI of 5∶1. **(** ***A*** **)** Average number of bacteria present per cell after 5 hr of infection. **(** ***B*** **)** Percentage of bacteria that did not co-localize with LAMP-1 and/or Rab5 and, therefore considered to be in the cytosol, among the total number of bacteria at different time points after infection. Representative confocal images shown for the 72 hr time point after infection indicate that *M. bovis* BCG is completely co-localized with **(** ***C*** **)** LAMP-1 (red) or **(** ***D*** **)** Rab5 (red) but that some of the H37Rv organisms do not. **(** ***E*** **)** Infected cells were permeabilized with digitonin and stained with rabbit anti-Mtb antibody followed by anti-rabbit IgG-Alexa 594 (red). The *M.tb* organisms in the cytosol accessible to these antibodies stained red and yellow after merge, respectively. Bacteria that stained green in merged pictures were localized in the phagosome or phagolysosome. The nucleus of the cells was stained with DAPI (blue). The upper row of each section shows macrophages infected with H37Rv and the lower row shows macrophages infected with *M. bovis* BCG. **(** ***F*** **)** Kinetics of bacterial translocation to the cytosol of infected macrophages from 0 to 96 hr after infection. This was calculated from confocal studies with digitonin-permeabilized cells, as in panel **(** ***E*** **)**. Bacteria in the cytoplasm of macrophages were counted from an average of 50 infected cells, which were accessible to the anti-Mtb antibody (red) and turned yellow after merging pictures. Experiments were run in triplicates and repeated three times. Representative data are shown.

Previous studies demonstrated that *M.tb* evolved mechanisms for inhibiting phagosome-lysosome fusion [1], [17], and to neutralize the acidic environment of the phagolysosomal compartment [1] to ensure unhindered survival and growth within phagocytes. However, recently it has been shown that *M.tb* successfully evades phagolysosomes and translocates to the cytosol in DCs [5]. DCs are neither the primary cell of *M.tb* replication, nor do they produce nitric oxide (NO) and reactive oxygen intermediates (ROI) that mediate bactericidal activities. In contrast, a small fraction of endocytosed *M. marinum*, a virulent *Mycobacterium* species in fish, translocates to the cytosol of macrophages [6]–[8]. Thus, we evaluated the capacity of *M.tb* to translocate to the cytosol in primary murine macrophages. We infected thioglycollate-elicited macrophages with *M.tb* or BCG organisms labelled with the green fluorescent dye FITC. Within four hours both H37Rv and BCG were taken up by the macrophages in similar numbers (**Fig. 1** ***A***). To identify intracellular organelles, we permeabilized cells and stained them with antibodies against organelle-specific markers. Cells were stained for the endosomal marker Rab5 and the lysosomal marker LAMP-1 at different times and analyzed by confocal microscopy. We found that during the first 24 hr almost all bacteria co-localized with either Rab5 or LAMP-1 or both, suggesting that they were present in either phagosomal or phagolysosomal compartments. Thereafter, some of the H37Rv organisms no longer co-localized with either Rab5 or LAMP-1 or both, suggesting that they might have escaped from the intracellular compartment to the cytosol (**Fig. 1** ***B*** *-* ***D***). However, almost all BCG organisms remained co-localized with Rab5 and LAMP-1, suggesting that they remained within the phagolysosomal compartment (**Fig. 1** ***B***–***D***). By 96 hr, around 30% of the intracellular H37Rv had escaped the phagolysosome and were found in the cytosol, whereas very few, if any BCG were found in the cytosol (**Fig. 1** ***B***). To further confirm that these organisms were indeed in the cytosol, we permeabilized infected cells with digitonin and stained them with antibody against the cell wall of *M.tb* to detect the cytosolic compartment as described by Collins and co-workers [15], [16]. Interestingly, we found that after 72 hr, a population of H37Rv organisms were stained with specific antibody indicating their presence in the cytosol, whereas BCG inside the phagosome was not accessible to the antibody and remained within phagolysosomes (**Fig. 1** ***E***). As a positive control, we treated these digitonin permeabilized infected macrophage with 0.2% tritonX-100, and we found that both H37Rv and BCG were equally stained with M.tb specific antibody (data not shown). We further analyzed the kinetics of appearance of *M.tb* in the cytosol using this method. We found that the kinetics of *M.tb* entry into the cytosol obtained using this method was very similar to that obtained with the Rab-5/LAMP-1 exclusion method. Similar to the findings obtained with the Rab-5/LAMP-1 exclusion method, we did not detect BCG organisms in the cytosol using specific antibody staining in digitonin-permeabilized and infected macrophages (**Fig. 1** ***F***). Therefore, these observations suggested that translocation from the phagolysosome to the cytosol is a property of virulent *M.tb* strains.

### ESAT-6, a gene product of the RD-1 region, plays an important role in transmigration of *M.tb* to the cytosol

**Figure 2:**
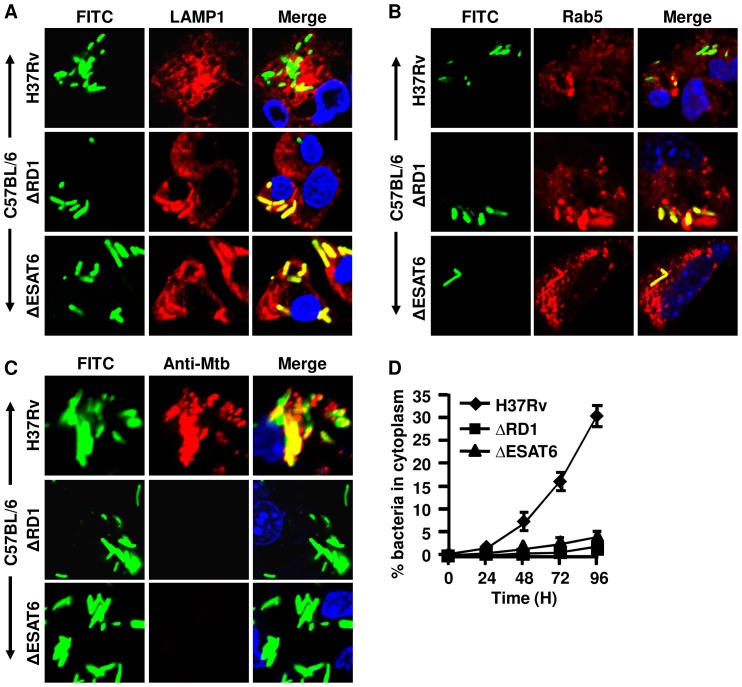
*Mycobacterium tuberculosis* H37Rv mutants ΔESAT6 and ΔRD1 fail to enter the cytosol of wild type (C57BL/6) macrophages. Bacteria were labelled green with FITC and used to infect peritoneal macrophages from C57BL/6 mice at an MOI of 5∶1. The average number of bacteria present per cell after 5 hr of infection was the same. Representative confocal images show data at the 72 hr time point after infection. *M.tb* mutants H37RvΔRD1 and H37RvΔESAT6 (green) are completely co-localized (yellow after merge) with (***A***) LAMP-1 (red) or (***B***) Rab5 (red), but some of the H37Rv organisms do not co-localize with these markers. (***C***) Infected cells were permeabilized with digitonin and stained with rabbit anti-Mtb antibody followed by anti-rabbit IgG-Alexa 594 (red). The *M.tb* in the cytosol accessible to these antibodies stained red or yellow after merging of pictures. Bacteria that were green in merged pictures are localized in phagolysosomes. The nucleus of the cells was stained with DAPI (blue). The upper row of each section shows H37Rv, the middle row shows H37RvΔRD1 and the lower row shows H37RvΔESAT6. (***D***) Kinetics of bacterial translocation to the cytosol of macrophages from 0 to 96 hr after infection. This was calculated from confocal studies of digitonin-permeabilized cells, as in panel (***C***). Bacteria in the cytoplasm of macrophages were counted from an average of 50 infected cells, which were accessible to the anti-Mtb antibody (red) and turned yellow after merging pictures. Data showed that around ∼30% of H37Rv (♦) bacteria escaped to the cytosol after 96 hr, whereas ΔRD1 (▪) and ΔESAT6 (▴) were unable to enter the cytoplasm. Experiments were run in triplicates and repeated thrice. Representative data are shown.

In the previous section we demonstrated that the virulent *M.tb* strain H37Rv but not the avirulent *M. bovis* BCG strain could translocate from the phagolysosome into the cytosol. Therefore, transmigration to the cytosol might be linked to virulence of *M.tb*. As compared with *M.tb*, BCG possesses deletions of multiple genomic segments, which are called regions of difference (RD). RD-1 is the most prominent deletion and has been linked to the loss of virulence in BCG [9], [18]. Notably, an H37Rv strain with a deletion of RD-1 resembles BCG in many biological functions, including infectivity and survival in the host [9]. Therefore, we examined whether the ΔRD-1 mutant of H37Rv can translocate from the phagolysosome to the cytosol. H37Rv and H37RvΔRD-1 exhibited similar capacity to infect macrophages (data not shown). As expected, we found that ΔRD-1 resembled BCG in that they were both unable to migrate to the cytosol as seen by LAMP-1 and Rab5 colocalization in confocal studies (**Fig. 2** ***A***–***B***), antibody accessibility assays in digitonin-permeabilized macrophages (**Fig. 2** ***C***). In the confocal studies with digitonin-treated cells, we found that around 30-35% of the intracellular H37Rv had escaped from the phagosome to the cytosol after 96 hr, whereas H37RvΔRD-1 was not observed in the cytosol (**Fig. 2** ***D***). Therefore, we concluded that the RD-1 region plays an important role in the transmigration of H37Rv from phagolysosomes to the cytosol.

Within the RD-1 region, ESAT-6 and CFP-10 are two prominent proteins that form a complex that plays a role in protein secretion in mycobacteria [18], [19]. Akin to the ΔRD-1 mutant, a deletion mutant of virulent *M.tb* in ESAT-6 (ΔESAT-6) resembles BCG in its biological functions, and has been shown to play an important role in mycobacterial virulence [20], [21]. Furthermore, ESAT-6 has been shown to play a role in the translocation of *M. marinum* organisms from the phagolysosome to the cytosol [8]. Therefore, we next performed experiments with H37RvΔESAT-6. Similar to ΔRD-1, ΔESAT-6 bacteria were unable to translocate to the cytosol (**Fig. 2** ***A***–***D***). Therefore, RD-1 and ESAT-6 are critical for transmigration of virulent *M.tb* from the phagolysosome into the cytosol of macrophages.

### TLR-mediated signals are required for confining mycobacteria within phagolysosomes

**Figure 3:**
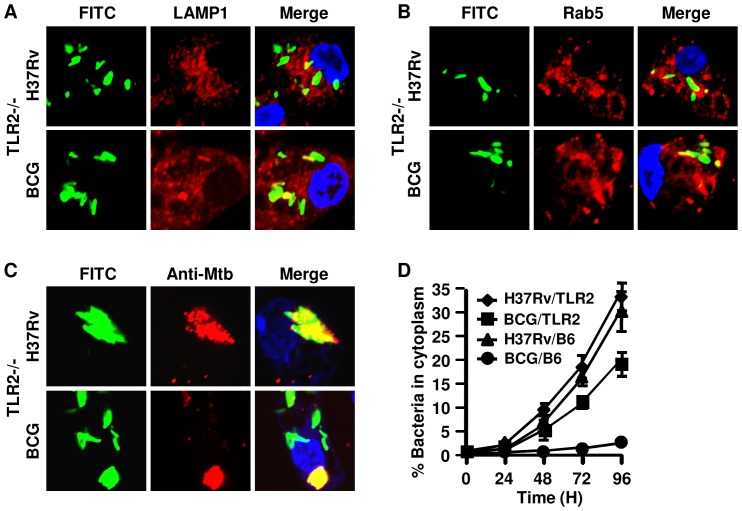
TLR-2-deficient macrophages fail to restrict *M. bovis* BCG to phagolysosomes. Bacteria were labelled green with FITC and used to infect peritoneal macrophages from TLR-2^−/−^ or wild type C57BL/6 mice at an MOI of 5∶1. The average number of bacteria present per cell after 5 hr of infection was the same. Representative confocal images show data at the 72 hr time point after infection in macrophages. In cells from TLR-2^−/−^ mice, a population of H37Rv and *M. bovis* BCG organisms are not co-localized (green after merging pictures) with either (***A***) LAMP-1 (red) or (***B***) Rab5 (red). (***C***) Infected cells were permeabilized with digitonin and stained with rabbit anti-Mtb antibody followed by anti-rabbit IgG-Alexa 594 (red). The *M.tb* organisms in the cytosol accessible to these antibodies stained red and yellow after merging pictures. Bacteria that were green in merged pictures were localized to phagolysosomes. The nucleus of the cells was stained with DAPI (blue). The upper row of each section shows H37Rv and the lower row shows BCG. (***D***) The kinetics of bacterial translocation to the cytosol of macrophages from 0 to 96 hr after infection. This was calculated from confocal studies of digitonin-permeabilized cells (♦ H37Rv- and ▪ BCG-infected macrophages from TLR-2^−/−^ mice; ▴ H37Rv- and • BCG-infected macrophage from C57BL/6 mice), as in panel (***C***). Bacteria in the cytoplasm of macrophages were counted from an average of 50 infected cells that were accessible to the anti-Mtb antibody (red) and turned yellow after merging pictures. Experiments were run in triplicates and repeated three times. Representative data are shown.

**Figure 4:**
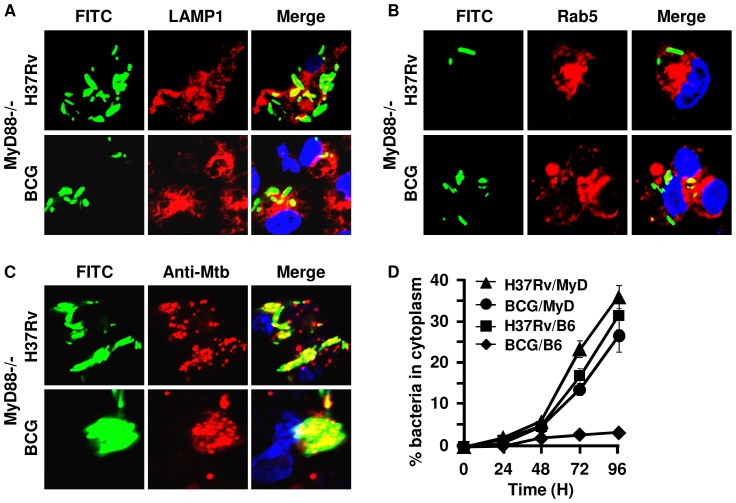
*M. bovis* BCG enters the cytosol in MyD88-deficient macrophages. Bacteria were labelled green with FITC and used to infect peritoneal macrophages from MyD88^−/−^ or wild type C57BL/6 mice at an MOI of 5∶1. The average number of bacteria present per cell after 5 hr of infection was the same. Representative confocal images are shown at the 72 hr time point after infection of macrophages. In macrophages from MyD88^−/−^ mice, a population of H37Rv and *M. bovis* BCG are not co-localized (green after merging pictures) with either (***A***) LAMP-1 (red) or (***B***) Rab5 (red). (***C***) Infected cells were permeabilized with digitonin and stained with rabbit anti-Mtb antibody followed by anti-rabbit IgG-Alexa 594 (red). The *M.tb* in the cytosol accessible to these antibodies stained red and yellow after merging pictures. Bacteria that were green in merged pictures are localized in phagosomes. The nucleus of the cells was stained with DAPI (blue). The upper row of each section shows H37Rv and the lower row shows BCG. (***D***) The kinetics of bacterial translocation to the cytosol of the macrophages from 0 to 96 hr after infection (▴ H37Rv- and • BCG-infected macrophages from MyD88^−/−^ mice; ▪ H37Rv- and ♦ BCG-infected in macrophages from C57BL/6 mice). This was calculated from confocal studies of digitonin-permeabilized cells, as in panel (***C***). Bacteria in the cytoplasm of macrophages were counted from an average of 50 infected cells that were accessible to the anti-Mtb antibody (red) and turned yellow after merging pictures. Experiments were run in triplicates and repeated three times. Representative data are shown.

Previously, we have shown that ESAT-6 binds to TLR-2 and induces production of TGF-β and other cytokines in macrophages [22]. Thus, we considered the possibility that engagement of TLR-2 with ESAT-6 plays a role in the translocation of phagocytosed *M.tb* to the cytosol. To test this hypothesis, we performed experiments with macrophages isolated from TLR-2^−/−^ mice. All mycobacterial strains (H37Rv, BCG, H37RvΔRD-1 and H37RvΔESAT-6) infected TLR-2^−/−^ macrophages at levels comparable to those isolated from wild type mice (data not shown). Surprisingly, each of these strains was able to successfully escape to the cytosol of macrophages derived from TLR-2^−/−^ mice (**Fig. 3** ***A*** **–** ***D***), irrespective of their virulence. Therefore, we concluded that TLR-2 is required for confining phagocytosed bacteria within phagolysosomes. To provide further support for this conclusion, we performed experiments with macrophages isolated from MyD88^−/−^ mice. Consistent with the studies with TLR-2^−/−^ macrophages, both virulent and mutant strains were successfully translocated to the cytosol of MyD88^−/−^ macrophages (**Fig. 4** ***A*** **–** ***D***). Therefore, we concluded that the TLR-2 - MyD88 pathway plays an important role in confining bacteria within the phagolysosome. This observation clearly suggested that, in the absence of TLR signaling, phagolysosomal maturation is defective, which is in agreement with a previous report using a different experimental system [12].

### Virulent *M.tb* inhibits innate immune signals to facilitate its translocation from the phagolysosome to the cytosol

**Figure 5:**
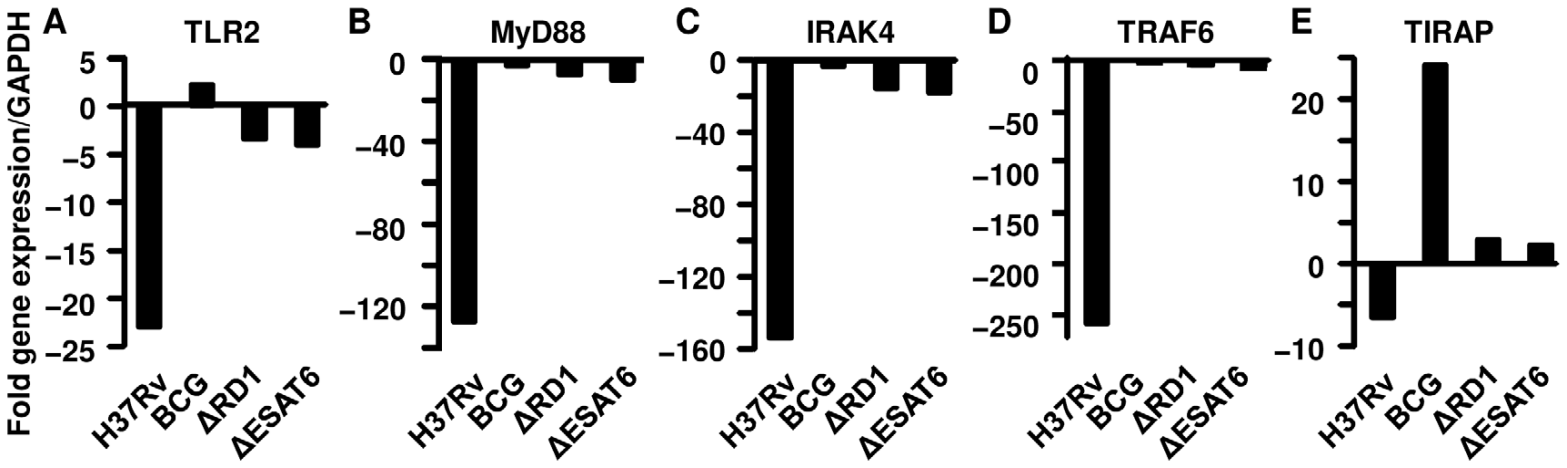
*Mycobacterium tuberculosis* H37Rv down-regulates the expression of key genes involved in the TLR-2 - MyD88 signaling pathway. Macrophages form wild type (C57BL/6) mice were infected with the indicated mycobacterial strains and 48 hr later cells were harvested for either analysis of relative mRNA expression of the indicated genes by quantitative RT-PCR or protein expression using immune-blotting. The gene expression of **(** ***A*** **)** TLR-2, **(** ***B*** **)** MyD88, **(** ***C*** **)** IRAK4, **(** ***D*** **)** TRAF6 and **(** ***E*** **)** TIRAP was down-regulated in macrophages infected with *M.tb* H37Rv. In contrast, no changes were observed in the expression of these genes in macrophages infected with BCG or the H37RvΔRD-1 and H37RvΔESAT6 mutants of H37Rv. The mRNA expression profiles were normalized with respect to expression of the GAPDH gene for each sample. The relative expression of genes in infected to uninfected macrophages is shown in each panel. Fold increase in the expression of each gene was calculated with respect to uninfected control at the same time point using the 2^−ΔΔCt^ method. Data shown here are representative of three independent experiments and qPCR assays were set up in triplicate for each target and the GAPDH gene.

In the previous sections we clearly showed that TLR-mediated signaling plays a critical role in confining avirulent bacteria within the phagolysosomal compartment. However, in wild type macrophages virulent bacteria can translocate to the cytosol, suggesting that virulent *M.tb* can interfere with TLR signaling to facilitate its escape from the phagolysosome. Therefore, we examined whether *M.tb* H37Rv can modulate TLR-mediated signaling events. For this purpose we evaluated the expression of TLR-2, MyD88, IRAK4, TRAF6, and TIRAP in macrophages at 48 hr after infection with H37Rv, BCG, H37RvΔRD-1 or H37RvΔESAT-6. We found that the expression of MyD88, IRAK4 and TRAF6 was down-regulated in macrophages infected with *M.tb* H37Rv, expression of TLR-2 was modestly down-regulated, and TIRAP expression was not affected (**Fig. 5** ***A–E***). No changes in the expression of any of these genes were observed in macrophages infected with BCG or the ΔRD-1 and ΔESAT-6 mutants (**Fig. 5** ***A–E***). These observations suggested that virulent *M.tb* strains modulate the expression of innate signaling molecules that control the entry of microorganisms into the cytosol. Additional studies will be needed to investigate the mechanisms involved.

## Discussion

It has been well documented that *M. marinum* translocates from the phagolysosome to the cytosol in macrophages [6]–[8], providing a possible explanation for its capacity to induce CD8^+^ T cell responses. Although, previous studies using transmission electron microscopy have provided some evidence for the presence of free *M.tb* organsims in the cytoplasm of macrophages [23]–[25], the mechanism has remained unclear. A large volume of work has indicated that *M.tb* survives within phagosomes or phagolysosomal compartments by inhibiting lysosomal fusion and neutralizing the acidic phagolysosomal environment, respectively [1]–[4], [17], [21], [24], [26]–[31]. However, it is well accepted that *M.tb* mounts CD8^+^ T cell responses, and that CD8^+^ T cells play an important role in host protection against TB [32], [33]. A recent elegant study by Van der wel and colleagues conclusively demonstrated that *M.tb* can translocate to the cytosol [5]. However, this study was mainly performed in DCs, which are neither the primary cell type where *M.tb* replicates, nor do they have antimicrobial defensive mechanisms such as reactive nitrogen and oxygen molecules. Therefore, we revisited these issues with primary macrophages. We investigated mycobacterial factors as well as host factors involved in the escape of *M.tb* organisms from phagolysosomal compartments in macrophages.

We found that up to 24 hr after infection nearly all mycobacterial strains, irrespective of their virulence status, remained in phagolysosomal compartments. However, some of the virulent H37Rv strains gradually transmigrated to the cytosol, reaching a maximum of 30% at 96 hr. While this result is in agreement with a previous study [5], we found that only a fraction of the organisms translocated to the cytosol. This apparent difference might be due to the use of macrophages in our study and DCs in the previous study [5]. However, earlier studies might have missed translocation of *M.tb* from phagolysosomal compartments to the cytosol in macrophages because most (70% or more) bacteria remained in phagolysosomal compartments.

Previous studies have indicated that genes within the RD1 region [8] of *M.tb*, *M. marinum*, *M. africanum*, *M. bovis* and *M. leprae* [34], [35], which partly encode for a type VII secretion system called ESX-1, play important roles in the cytosolic translocation of *M. marinum* [8]. We found that BCG, as well as H37RvΔRD-1 and H37RvΔESAT-6 were unable to translocate to the cytosol, suggesting that ESAT-6 plays an important role in transmigration of *M.tb* into the cytosol. RD1 is conserved between several species of virulent mycobacteria, and plays a role in lysis of host cells and spread of the microorganisms to uninfected macrophages [19]. Therefore, the ESX-1 secretory system, which is specialized to secrete CFP-10 and ESAT-6 in a heterodimeric form [34], [35], has an important role in the virulence of *M.tb* [9], [36], [37] and is required for phagosome escape and host cell lysis. Mutations within this region lead to attenuation of cytolytic activity, host cell lysis, and cell-to-cell spread of bacteria [8], [10], [38], [39]. ESAT-6 plays a role in the translocation of *M. marinum* organisms from the phagolysosome to the cytosol [8]. Importantly, prior studies have shown that ESAT-6 within the RD1 region and secreted through ESX-1 also plays a role in plasma membrane lysis, which facilitates spread of *M.tb* to uninfected macrophages [8], [10], [19], [38], [40].

An elegant study by Blander and Medzhitov provided evidence that TLR signaling is required for phagolysosome maturation in macrophages in response to *M.tb* infection [12]. It is also known that TLR2 recognizes *M.tb* components and induces proinflammatory signals [41]. Furthermore, our earlier studies showed that ESAT-6 binds with TLR-2, and this interaction facilitates cytokine production in macrophages [22]. Therefore, we tested whether TLR-2 plays a role in the translocation of *M.tb* to the cytosol. We observed that all mycobacterial strains, irrespective of their virulence status, successfully migrated to the cytosol of macrophages derived from TLR-2^−/−^ mice. To confirm that the TLR-2-MyD88 pathway plays a critical role in confining the microorganisms to phagolysosomes, we performed similar experiments with macrophages from MyD88^−/−^ mice and obtained similar results.

It is well established that gram-positive bacteria such as *M.tb* that are recognized through TLR-2 activate host cells through the TLR2 - MyD88 - IRAK4 - TRAF6 - NF-κB signal transduction pathway [42]. Our results indicated that the TLR-2-MyD88 pathway plays an important role in confining mycobacteria within phagolysosomal compartments. Nevertheless, virulent *M.tb* strains successfully migrate to the cytosol. Therefore, we examined whether *M.tb* subverts molecules within this innate immune signaling pathway. We observed that *M.tb* downregulates TLR2, MyD88, IRAK4 and TRAF6 mRNA expression, but we found no significant effect on TIRAP mRNA expression (**Fig. 5** ***A–E***). This is consistent with earlier findings indicating that MyD88^−/−^ mice are highly susceptible to *M.tb* infection, whereas TIRAP expression is dispensable for the early response to acute *M.tb* infection [43]. In summary, we have provided strong evidence that virulent *M.tb* has evolved mechanisms to escape from the phagolysosomal compartment to the cytosol in macrophages. The TLR-2-MyD88 signaling axis appears to play an important role in confining phagocytosed bacteria within the phagolysosomal compartment. The virulent *M.tb* strain H37Rv modulates the expression of TLR-2 and successfully inhibits the expression of TLR-2-mediated signaling molecules, thus facilitating escape of the microorganisms to the cytosol. Transmigration to the cytosol permits virulent strains of *M.tb* to sequester themselves from the hostile microenvironment of the phagolysosome to facilitate their continued survival and growth within susceptible hosts.
